# αvβ3 Integrin as a Link between the Development of Fibrosis and Thyroid Hormones in Systemic Sclerosis

**DOI:** 10.3390/ijms24108927

**Published:** 2023-05-18

**Authors:** Maia Yamila Kohon, Mor Zaaroor Levy, Tzipi Hornik-Lurie, Avshalom Shalom, Ariel Berl, Liat Drucker, Yair Levy, Shelly Tartakover Matalon

**Affiliations:** 1 Sackler Faculty of Medicine, Tel Aviv University, Tel Aviv 6997801, Israelmor.zaaroor@gmail.com (M.Z.L.); druckerl@clalit.org.il (L.D.); levy.yair@clalit.org.il (Y.L.); 2 Autoimmune Research Laboratory, Meir Medical Center, Kfar Saba 4428164, Israel; 3 Data Research Department, Meir Medical Center, Kfar Saba 4428164, Israel; tzipi.hornik@clalit.org.il; 4 Department of Plastic Surgery, Meir Medical Center, Kfar Saba 4428164, Israel; arielberl23@gmail.com; 5 Oncogenetics Laboratory, Meir Medical Center, Kfar Saba 4428164, Israel; 6 Department of Internal Medicine E, Meir Medical Center, Kfar Saba 4428164, Israel

**Keywords:** systemic sclerosis, fibrosis, ECM, myofibroblast, integrin αvβ3, thyroid hormones

## Abstract

Systemic sclerosis (SSc) is an autoimmune disease characterized by fibrosis of the skin and internal organs. Key players mediating fibrosis are myofibroblasts (MF) that, following transforming growth factor β (TGFβ) exposure, produce a collagen-rich extracellular matrix (ECM) that induces myofibroblast differentiation. Myofibroblasts express αvβ3 integrin (a membrane receptor for thyroid hormones) and miRNA-21 that promotes deiodinase-type-3 expression (D3), causing the degradation of triiodothyronine (T3) that attenuates fibrosis. We hypothesized that αvβ3 affects the fibrotic processes through its thyroid hormones (THs) binding site. To test this, dermal fibroblasts (DF) were cultured with/without TGFβ and removed with a base, leaving only normal/fibrotic ECMs in wells. Then, DF were cultured on the ECMs with/without tetrac (αvβ3 ligand, T4 antagonist), and evaluated for pro-fibrotic characteristics, αvβ3, miRNA-21, and D3 levels. Blood free-T3 (fT3), miRNA-21 levels, and the modified Rodnan skin score (MRSS) were evaluated in SSc patients. We found that the “fibrotic-ECM” significantly increased the pro-fibrotic characteristics of DF and the levels of miRNA-21, D3, and αvβ3, compared to the “normal-ECM.” Tetrac significantly inhibited the effects of the “fibrotic-ECM” on the cells. In accordance with tetrac’s effect on D3/miRNA-21, a negative correlation was found between the patients’ fT3 to miRNA-21 levels, and to the development of pulmonary arterial hypertension (PAH). We conclude that occupying the THs binding site of αvβ3 may delay the development of fibrosis.

## 1. Introduction

Systemic sclerosis (SSc) is an autoimmune disease characterized by aberrant immune activation, vascular injury, and extensive tissue fibrosis of the skin and internal organs [1]. Vascular injuries are present in various organs and include pulmonary arterial hypertension (PAH) and digital ulcers. These autoimmune attacks on blood vessels are accompanied by fibrosis, which is characterized by the accumulation of fibrous connective tissue, leading to tissue malfunction. The pursuit of effective anti-fibrotic treatments is ongoing [2,3,4]. Yet, the high disease burden with increased morbidity and mortality, together with the short-term, partial effectiveness of anti-fibrotic treatments, have created a high unmet need for novel therapeutic anti-fibrotic strategies in SSc [5]. Myofibroblasts (MF) are key players in the development of fibrosis [6]. They constitutively produce large amounts of extracellular matrix (ECM) and enzymes such as matrix metalloproteinase (MMPs) that degrade the ECM. Eventually, the increased ECM mass and its altered composition and organization lead to the development of fibrosis [7].

The fibrotic skin of SSc patients is characterized by an excess accumulation of fibronectin and collagen that stiffens the ECM [8,9]. MF differentiate from various cells, including fibroblasts [10]. They resemble fibroblasts, yet they also have muscle cell characteristics such as the expression of alpha-smooth muscle actin (αSMA) in their cytoplasm. The fibrotic ECM provides biological and mechanical cues that induce fibroblast proliferation, as well as elevated secretion of ECM and transforming growth factor β TGFβ) [6]. In a positive feedback loop, the ECM and TGFβ cause persistent fibroblast activation, driving a vicious cycle [8,11]. Finding a way to attenuate this vicious cycle may help in the development of a treatment for fibrosis.

TGFβ is a key growth factor involved in MF activation and their pro-fibrotic effects [1,10]. The pro-fibrotic effect of TGFβ is strongly mediated by SMAD3 phosphorylation [12]. Many mechanisms regulate TGFβ signaling and fibrosis. For example, the TGFβ pathway can be activated by miRNA-21, which downregulates SMAD7, a TGFβ pathway inhibitor [13]. The level of miRNA-21 is upregulated by TGFβ [13], creating a positive feedback loop. TGFβ and miRNA-21 may also affect fibrosis by indirectly upregulating deiodinase type-3 (D3) [14]. D3 is a plasma membrane enzyme that inactivates the thyroid hormone triiodothyronine (T3). D3 expression is increased by T3 and reduced in hypothyroidism [15]. The D3 target, T3, inhibits the TGFβ pathway and promotes anti-fibrotic effects through its binding to the nuclear thyroid hormone (TH) receptors [16]. Compared to healthy people, miRNA-21 levels are increased in fibroblasts and in the circulation of SSc patients, especially in the sera of patients with local SSc [13,17,18]. PAH is a common complication for individuals with local SSc. Furthermore, most of the TGFβ is bound to the ECM [19] and must be released from the ECM to become active. TGFβ is synthesized as a precursor protein composed of growth factor and latency-associated peptide (LAP). LAP contains the amino acid sequence arginine-glycine-aspartic (RGD motif). This complex is connected to the ECM by the latent TGFβ binding protein. TGFβ can be released from the ECM by proteases such as MMP-9 [20] and by αv integrins [8] that bind the LAP through its RGD recognition site and induce a conformational change [7,8,20]. One of the αv integrins whose level is highly expressed by MF is αvβ3 [21,22,23]. The β3 integrin subunit is upregulated in the fibroblasts of SSc patients [23]. Interestingly, αvβ3 integrin is also a cell surface receptor for the thyroid hormones (THs) T3, L-thyroxine (T4), and the T4 analogue and antagonist, tetraiodothyroacetic acid (tetrac) [24]. A non-genomic effect of THs mediated by αvβ3 integrin was previously demonstrated with various cells, including fibroblasts, cancer cells, and muscle cells [25,26]. The αvβ3 binding site for T4/T3/tetrac is located adjacent to the RGD recognition site. T4/T3 binding to the integrin is displaced by RGD and tetrac [24,27,28]. Moreover, various tetrac-initiated effects are attenuated by the RGD peptide [24,27,28]. This interplay suggests possible interactions and crosstalk between the THs binding site of αvβ3 to proteins that express RGD, such as fibronectin, and the LAP protein that is bound to TGFβ. Indeed, previous publications demonstrated a connection between THs, autoimmune diseases, and fibrosis [16,26,29,30,31,32]. Since high levels of αvβ3 integrin are expressed by MF in the fibrotic sites and the THs binding site of αvβ3 is close to the RGD recognition site, we hypothesized that ligand binding to the THs binding site of αvβ3 may interfere with and attenuate the fibrotic process.

The current study used a biological model to evaluate the effects of the ECM produced by MF on the naive dermal fibroblast (DF) phenotype and αvβ3 expression. Using this model, we showed that the “fibrotic-ECM” significantly increased the pro-fibrotic characteristics of DF and the levels of αvβ3, miRNA-21, and D3 compared to the “normal-ECM.” We used this model to evaluate the connection between the THs binding site of αvβ3 integrin and fibrosis-related pathways. This study demonstrated that tetrac significantly inhibited all the effects of the fibrotic-ECM on the cells. In accordance with tetrac’s effect on miRNA-21 and D3, we evaluated miRNA-21 and blood-free T3 (fT3) levels in SSc patients and found a negative correlation between fT3 and miRNA-21 levels, and between fT3 levels and the development of PAH. Our results demonstrate for the first time that manipulating the THs binding site of αvβ3 integrin affects pathways related to fibrosis and suggest that blocking this site with tetrac (αvβ3 ligand and T4 antagonist) may delay the development of fibrosis.

## 2. Results

### 2.1. Isolation and Characterization of DF

**Figure 1:**
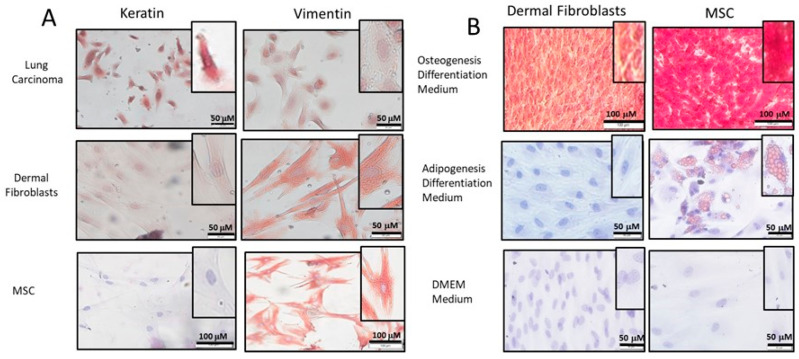
Characterization of dermal fibroblasts. (**A**) DF were isolated from the dermis and MSC from the subcutaneous fat. Cells were tested for vimentin (mesenchymal marker) and keratin (epithelial marker) expression using immunocytochemistry. Lung carcinoma cells (epithelial origin) and MSC (mesenchymal origin) served as controls. The nucleus was stained for hematoxylin. (**B**) DF and MSC were cultured in osteogenesis (top)/adipogenesis (middle) differentiation medium and stained to test adipocyte and osteoblast differentiation. Cells cultured in the usual DMEM growth medium were used as control (bottom). Photomicrographs of the cells at ×100 (bottom panel in (**A**) and top panel in (**B**)) and ×200 (all other photos) magnifications were done using an inverted microscope.

DF were isolated from skin tissue. Following the adhesion of cells to culture dishes, ICC was used to show mesenchymal origin by the expression of vimentin (mesenchymal marker) and the lack of keratin expression (epithelial marker, Figure 1A). MSC isolated from subcutaneous adipose tissue and a lung carcinoma cell line were used as positive controls for cells from mesenchymal and epithelial origins, respectively (Figure 1A). Since MSC grow under the same conditions as fibroblasts, the isolated cells might have been heterogeneous and included both fibroblasts from the skin and MSC from the subcutaneous fat that express many similar markers. Yet, MSC are multipotent cells [33], while adult fibroblasts are not. Culturing the isolated cells and MSC in conditions suitable for differentiation of fibroblasts, adipocytes, or osteoblasts showed that our DF did not differentiate into adipocytes and osteocytes, while MSC did (Figure 1B), which validated the DF identity of our cells.

### 2.2. Differentiation of DF into MF

**Figure 2:**
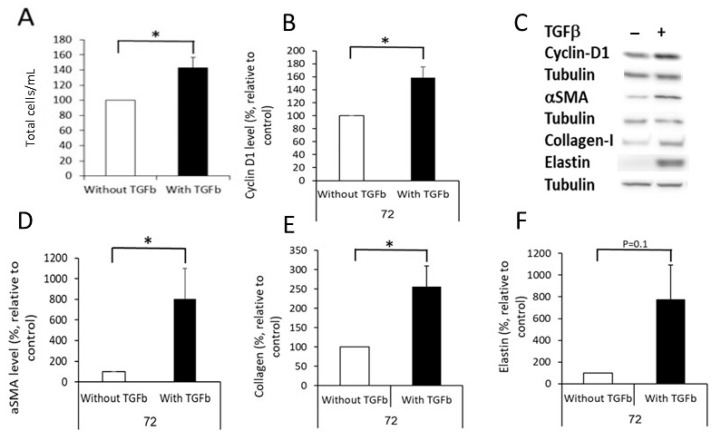
Differentiation of dermal fibroblasts into myofibroblasts. DF (100,000 cells/well) were cultured with or without TGFβ (TGFb in the graphs, 10 ng/mL) for 72 h. Then, cells were harvested and counted, proteins extracted, and the levels of cyclin-D1, αSMA, collagen-I, and elastin measured using Western blot. (**A**) describes the average number of cells at the end of the experiment (mean + SD). (**B**,**D**–**F**) show graphs of the average levels of cyclin-D1, αSMA, collagen-I, and elastin in cells exposed or not exposed to TGFβ for 72 h. (**C**) shows representative Western blot images of protein levels. * The results are significantly different (*p* ≤ 0.05, *n* = 5–6).

To induce DF differentiation into MF, we exposed them to TGFβ (10 ng/mL) for 72 h. Validation of differentiation was shown by the following: (1) increased cell proliferation (increased cell number, Figure 2A, *p* ≤ 0.05) and the level of the cell cycle regulator cyclin-D1 (Figure 2B,C, *p* ≤ 0.05); (2) increased levels of αSMA (Figure 2D, *p* < 0.05), collagen-I (Figure 2E, *p* ≤ 0.05), and elastin (Figure 2F, *p* = 0.1).

### 2.3. Preparation of Extracellular Matrices (ECM)

**Figure 3:**
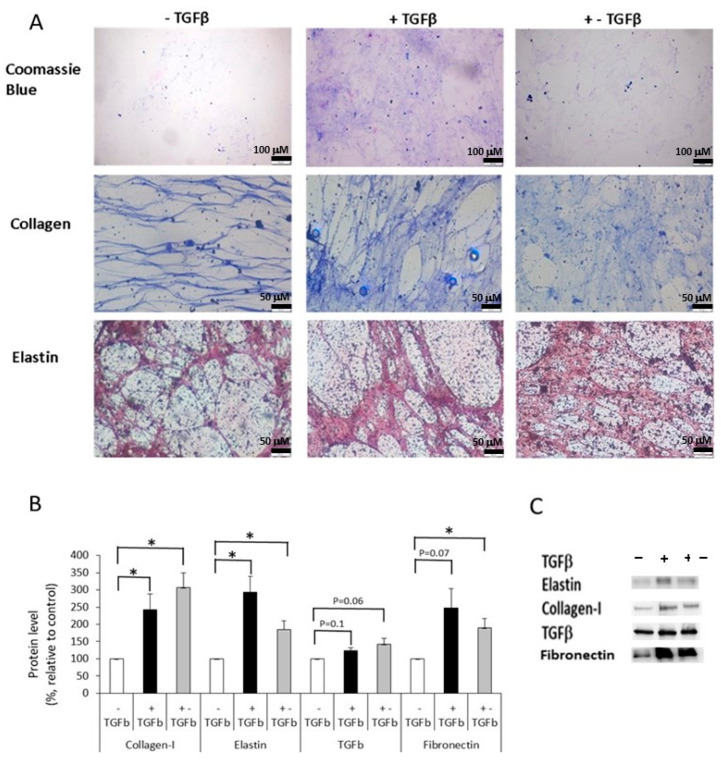
Preparation of extracellular matrices. DF (100,000 cells/well) were cultured as follows: 1. Without TGFβ (−TGFβ for 4 days); 2. With TGFβ (+TGFβ for 4 days); 3. One day with TGFβ (to trigger DF differentiation) and then 3 days without exogenous TGFβ (+−TGFβ). Next, cells were removed from wells using a strong base (NH_4_OH) and one of the following was undertaken: (1) the ECM was stained with Coomassie blue (protein dye), aniline acid (collagen dye), and Orcein (elastin dye). Then, photomicrographs of the ECM ×100 (Coomassie staining) and ×200 (all other photos) were taken using an inverted microscope. (2) The ECMs were collected to measure protein levels (collagen-I, elastin, fibronectin, and TGFβ) with Western blot. (**A**) shows representative photomicrographs of matrices stained with different dyes. (**B**) shows graphs of the protein levels in fibrotic-ECM (+TGFβ/+−TGFβ) and normal-ECM (−TGFβ) models. (**C**) shows representative Western blot images of protein levels. * The results are significantly different (*p* ≤ 0.05, *n* = 5–8).

Next, we wanted to produce and characterize the ECM secreted by naive DF cultured with or without TGFβ. These were termed fibrotic-ECM and normal-ECM, respectively. To produce the fibrotic-ECM, DF were either exposed to exogenous TGFβ throughout the entire period of ECM formation (marked as “+TGFβ”), or for one day only, and then transferred to new wells where they continued to secrete ECM without the addition of exogenous TGFβ (marked as “+−TGFβ”). The +−TGFβ model demonstrated the ability of cells differentiated from DF (by initial exposure to TGFβ) to maintain the differentiation process in an autologous manner. After culturing DF with/without TGFβ, the cells were removed using a strong base, leaving only the ECM in the wells. We characterized the ECM and found that those produced from DF that were exposed to exogenous TGFβ (+TGFβ) during its production had the strongest staining with Coomassie blue (Figure 3A), suggesting they secreted higher amounts of proteins. DF that were exposed to exogenous TGFβ for only one day before ECM production (+−TGFβ) stained less than the ECM described earlier, but more than the ECM produced by DF cultured without TGFβ (−TGFβ, Figure 3A). Furthermore, both fibrotic-ECMs (+TGFβ, +−TGFβ) had stronger staining for collagen and elastin than the normal-ECM (Figure 3A). Next, we collected the ECM and measured the levels of collagen-I, elastin, fibronectin, and TGFβ. In agreement with the histochemical staining, the levels of collagen-I, elastin, and fibronectin were significantly higher in the fibrotic-ECMs (+TGFβ, +−TGFβ) compared to their levels in the normal-ECM (−TGFβ). Similarly, a trend toward increased levels of TGFβ was found in the fibrotic-ECM compared to the normal-ECM (Figure 3B,C).

### 2.4. The Effect of Normal and Fibrotic-ECM on Naive DF

**Figure 4:**
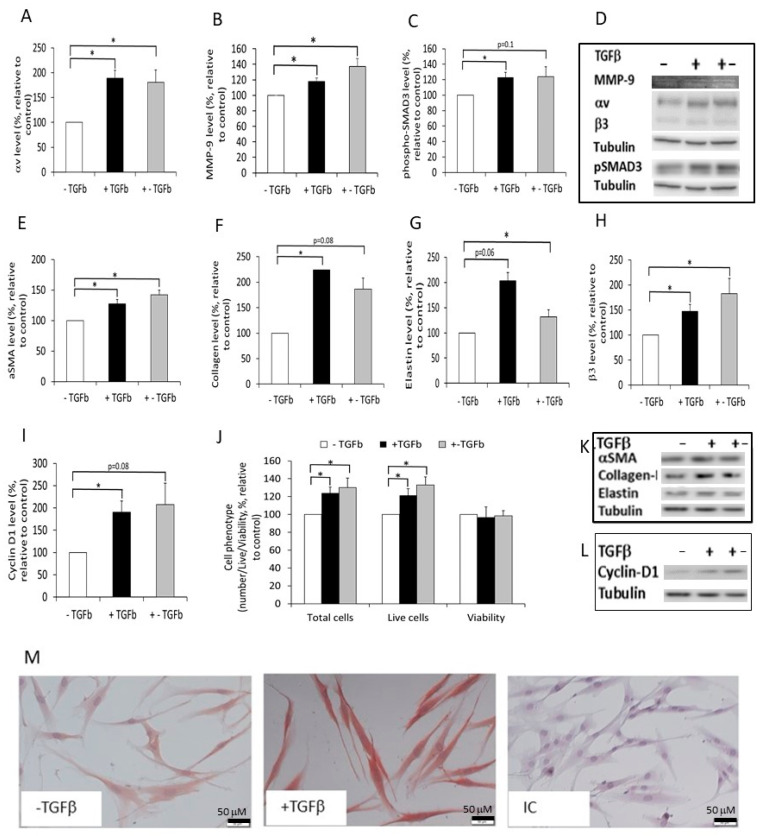
The effect of normal or fibrotic-ECM on naive DF: DF (100,000 cells/well) were cultured on normal (−TGFβ) or fibrotic-ECMs (+TGFβ, +−TGFβ) for 45 min or 48–72 h (TGFb in the graphs). Then, one of the following procedures was performed: (1) the secretomes were collected for evaluation of MMPs activity (48 h, (**B**)); (2) the cells were harvested and the proteins were extracted from the cells either after 45 min to evaluate the level of pSMAD3 (**C**), or after 48 h to evaluate the levels of αv (**A**), αSMA (**E**), collagen-I (**F**), elastin (**G**), β3 (**H**), and cyclin-D1 (**I**) with Western blot; (3) cells were counted using an automatic cell counter and trypan-blue (72 h, (**J**)); or (4) the cells were stained for αvβ3 using ICC (**M**). (**D**) shows representative zymogram gel of MMP9. (**D**,**K**,**L**) contain representative Western blots images of αv, β3, pSMAD, and tubulin (**D**), αSMA, collagen-I, elastin, and tubulin (**K**), and cyclin-D1 and tubulin (**L**) in the different treatments. (**M**) shows photomicrographs of DF cultured on normal (−TGFβ) or fibrotic (+TGFβ) stained with anti-αvb3 or isotype-matched control antibodies, (×200). The nucleus was stained with hematoxylin * The results are significantly different (*p* < 0.05, *n* = 5).

Both fibrotic models demonstrated that exposing DF to TGFβ, even for one day, induced the production of an ECM with more fibrotic characteristics. Next, we evaluated the effect of the fibrotic-ECMs produced by the cells on naive DF. We demonstrated that the fibrotic-ECMs contain increased levels of TGFβ (Figure 3B,C). Therefore, we questioned whether the DF that were cultured on the different ECMs developed tools to release the TGFβ bound to the matrices. Our results demonstrated a significant increase in αv levels and MMP-9 activity in DF cultured on both fibrotic-ECMs compared to DF cultured on normal-ECM (Figure 4A,B,D). These results support the notion that DF cultured on fibrotic-ECM have the potential to release the TGFβ from the ECM. Furthermore, we examined the effect of the fibrotic-ECMs on the activation of the TGFβ pathway in DF. To test this, we cultured DF on the different ECMs and evaluated the cellular levels of phosphorylated-Smad3 (pSMAD3). The results showed increased levels of pSMAD3 in DF cultured for 45 min on fibrotic-ECMs (+TGFβ, +−TGFβ) compared to those cultured on normal-ECM (−TGFβ, Figure 4C,D, *p* < 0.05 and *p* = 0.1, respectively). The TGFβ pathway regulates the expression of the MF biomarkers; αSMA, collagen-I, and elastin. Thus, we continued to examine the effect of the different ECMs on their expression. Results showed increased expression of those proteins (Figure 4E–G,K, increased by 27–123%) in cells cultured on fibrotic-ECMs compared to cells cultured on normal-ECM. These findings suggest that the fibrotic-ECMs induced DF differentiation into MF. Accordingly, the fibrotic-ECMs (+TGFβ, +−TGFβ) also increased the total cell number and the number of live DF (Figure 4J), compared to the control (−TGFβ) [6]. This increase in cell number may be a result of increased cell proliferation or decreased cell death. The cell count results demonstrated that there was no change in the viability of the cells (Figure 4J), suggesting that the higher number of cells found in DF cultured on fibrotic-ECMs reflect increased cell proliferation. In agreement with this, DF cultured on fibrotic-ECMs expressed elevated cyclin-D1, which is an essential kinase for cell cycle, compared to DF cultured on the normal-ECM (*p* < 0.05 and *p* = 0.08 in the +TGFβ and +−TGFβ models, respectively, Figure 4I,L).

### 2.5. The Effect of Normal and Fibrotic-ECM on DF αvβ3 Expression

Next, we examined whether DF cultured on fibrotic-ECMs also expressed higher levels of β3 integrin and found that on both fibrotic-ECMs models, DF expressed significantly increased β3 levels compared to DF cultured on normal-ECM (Figure 4H,D). Furthermore, we used ICC to stain DF cultured on normal/fibrotic-ECMs with anti αvβ3 antibodies to demonstrate that the full integrin (composed of αv + β3 chains) is expressed in the cells (Figure 4M).

The finding that DF treated with soluble TGFβ secrete fibrotic-ECM, which contains embedded TGFβ, and that this fibrotic-ECM continuously activates and differentiates naive DF into MF, demonstrates the existence of a positive feedback loop. This positive feedback loop contributes to the development of fibrosis in SSc. Therefore, interrupting this loop may delay the development of fibrosis.

### 2.6. The Effect of Tetrac on the Response of DF to Fibrotic-ECM

**Figure 5:**
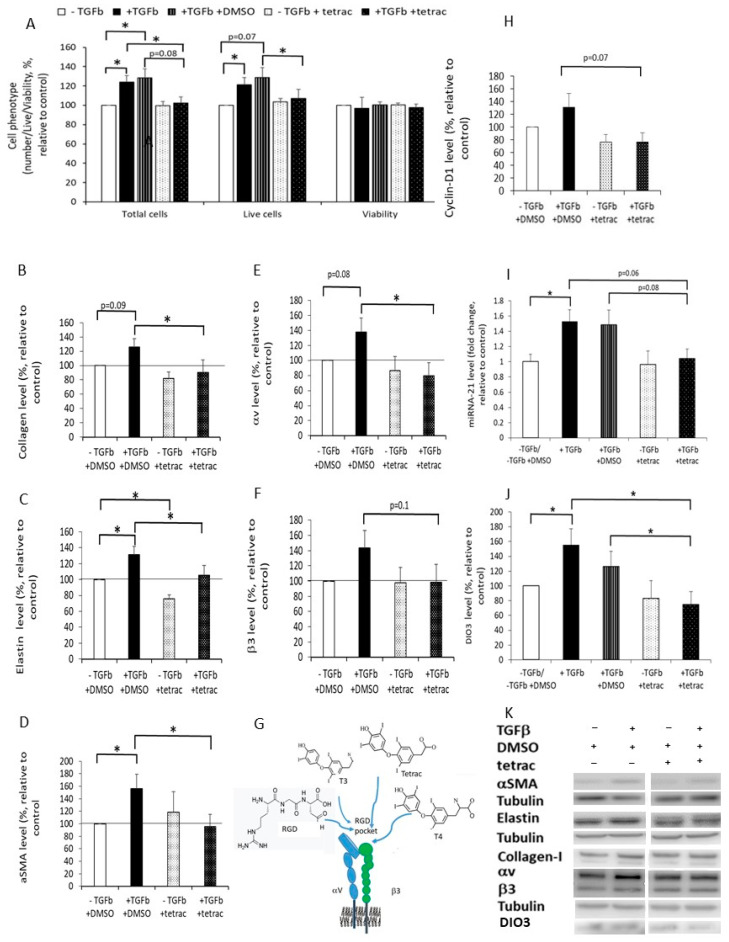
The effect of fibrotic-ECM and tetrac on DF cell proliferation and death and on the levels of MF biomarkers, αvβ3, miRNA-2,1 and D3: DF (100,000 cells/well) were cultured on normal (−TGFβ (TGFb in the graphs)) or fibrotic-ECM (+TGFβ) with/without 0.5 μM tetrac or its solvent (DMSO-KOH propylene glycol) for 48 h. Then, cells were harvested, and the following was undertaken: (1) the cells were counted using a counter and trypan-blue; (2) the proteins were extracted from the cells, and protein levels were measured by Western blot; and (3) the RNA was extracted from the cells, and the level of miRNA-21 was evaluated using qPCR. (**A**,**H**) shows the average number of total cells, live cells, viability, and cyclin-D1 at the end of the experiment (mean + SD). (**B**–**F**,**I**,**J**) show collagen-I (**B**), elastin (**C**), αSMA (**D**), αv (**E**), β3 (**F**), miRNA-21 (**I**), and D3 (**J**) levels, in cells cultured on ECMs with/without tetrac. (**G**) shows a schematic demonstration of the αvβ3 binding site of RGD, T3, T4 and tetrac. (**K**) shows representative Western blot images of protein levels in the different treatments. The blots contain skipping lanes. * The results are significantly different (*p* ≤ 0.05, *n* = 4–6).

T4, T3, and tetrac occupy sites in the integrin RGD binding pocket [34] (Figure 5G). Therefore, we speculated that the capture of αvβ3 by tetrac (M_W_ > 747 g/mol) may interfere with RGD binding (M_W_ = 346 g/mol), or the downstream response to RGD binding and may attenuate the differentiation of DF to MF and the fibrotic process. The fibrotic-ECM in our models increased the expression of αvβ3 levels in DF and induced their differentiation to MF. Therefore, we wanted to examine whether tetrac affects DF differentiation to MF. Our research model used a primary cell culture that is characterized by great variability. The variability in results was higher in the +−TGFβ model than in the +TGFβ model. This is probably because the +−TGFβ model was stimulated with TGFβ only initially, while the continuation of the experiment was influenced by the individual from whom the cells were taken. For this reason, we continued the study using only the +TGFβ model. The addition of 0.5 μM tetrac to DF cultured on normal-ECM (−TGFβ +tetrac) had no effect on cell number or viability compared to cells cultured on normal-ECM without tetrac (Figure 5A,H). However, decreased total and live cell numbers, and cyclin-D1 levels were observed in DF cultured on fibrotic-ECM with tetrac (+TGFβ +tetrac) compared to DF cultured on fibrotic-ECM without tetrac (+TGFβ +DMSO; Figure 5A,H). This inhibition of cell proliferation returned the number of DF cultured on fibrotic-ECM to that of DF cultured on normal-ECM. Furthermore, addition of tetrac to DF cultured on fibrotic-ECM (+TGFβ +tetrac) decreased the levels of the MF biomarkers; αSMA, collagen-I, and elastin, and the level of αvβ3, compared to DF cultured on fibrotic-ECM without tetrac; (+TGFβ +DMSO, for αSMA, collagen-I, elastin, and αv, *p* ≤ 0.05, for β3 *p* = 0.1 Figure 5B–F,K). In contrast, tetrac had no significant effect on the levels of these proteins except for elastin in DF cultured on normal-ECM (−TGFβ +tetrac compared to −TGFβ +DMSO).

### 2.7. The Effect of Fibrotic-ECM and Tetrac on miRNA-21 and D3 Expression

We found that αvβ3 integrin is involved in DF differentiation to MF, which is triggered by the TGFβ pathway. Since there is crosstalk between TGFβ and miRNA-21 and between them and D3, we speculated that αvβ3 integrin might also affect fibrosis by regulating miRNA-21 and D3 levels and thus the level of the anti-fibrotic hormone T3. To test this theory, we first evaluated whether DF cultured on fibrotic-ECM express higher levels of miRNA-21 and D3. We found significantly increased levels of both in cells cultured on fibrotic-ECM (+TGFβ) compared to cells cultured on normal-ECM (−TGFβ; Figure 5I–K). Next, we examined whether the addition of tetrac would inhibit these events. Adding tetrac downregulated miRNA-21 and D3 levels in DF cultured on fibrotic-ECM (+TGFβ +tetrac) compared to DF cultured on fibrotic-ECM without tetrac (+TGFβ +DMSO; Figure 5I–K), and the levels of miRNA-21 and D3 reached that of DF cultured on normal-ECM (−TGFβ +DMSO). In contrast to the effect of tetrac on DF cultured on fibrotic-ECM, it had no significant effect on miRNA-21 and D3 levels in DF cultured on normal-ECM (−TGFβ +tetrac compared to −TGFβ +DMSO; Figure 5I–K).

Our results demonstrated that tetrac may inhibit fibrosis by preventing DF differentiation to MF and therefore the production of the fibrotic-ECM, by decreasing miRNA-21 and D3 expression, demonstrating the potential to prevent the inactivation of the anti-fibrotic hormone T3, and by reducing αvβ3 expression. This suggests that tetrac may stop the vicious cycle between the fibrotic-ECM and the MF.

### 2.8. Analyzing miRNA-21 and fT3 Levels in SSc Patients’ Sera

Subsequent to the results that demonstrated a link between αvβ3, miRNA-21, and D3 levels (the T3 degradation enzyme) in the in vitro model, we examined whether there is also a link between miRNA-21 and fT3 levels in the circulation of SSc patients. To test this, circulating fT3 and miRNA-21 levels were evaluated, and the modified Rodnan skin score (MRSS) values of 18 SSc patients were obtained from their medical records. The MRSS evaluates skin thickness and is used as an outcome measure in SSc [35]. Seven of the SSc patients also had PAH. The patients were divided into groups according to their blood fT3 levels (high T3 or low T3). Since we found that the fT3 levels in SSc patients with PAH were also low (Table 1), we examined them as a separate group and as part of the group with low fT3 levels. The data is presented in Table 1**.**

**Figure 6:**
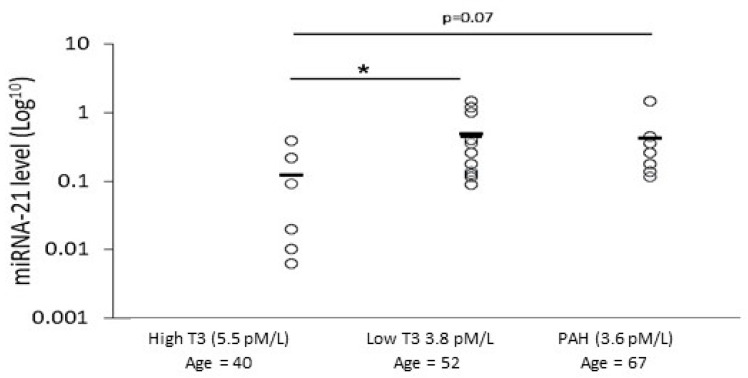
Evaluating fT3 and miRNA-21 levels in patients’ sera. Sera were collected from SSc patients with/without PAH, and analyzed for the levels of fT3 and miRNA-21. The figure shows miRNA-21 levels of sera from all patients in the three groups: low T3 (with/without PAH (*n* = 12), high T3 (*n* = 6), and PAH (low T3) (*n* = 7). The average fT3 level and age in each group appears next to the group name on the *x*-axis. * The results are significantly different (*p* ≤ 0.05).

In agreement with the in vitro results, a moderately negative correlation was found between miRNA-21 and fT3 levels in the patients (Table 2). fT3 expression in most of the patients was in the normal range (3.1–6.8 pmol/L) but 15/18 patients were below average. For the sub-group of patients, we defined fT3 values as high or low relative to the average fT3 values of all patients ($\tilde{x}$ = 4.3 pmol/L). miRNA-21 levels were compared between the groups. This analysis supported the previous negative correlation between miRNA-21 and fT3 presented in Table 2 and showed that miRNA-21 levels in the low T3 group were significantly higher than in the high T3 group (fold change 3.4, *p* ≤ 0.05; Figure 6).

The PAH patients had low fT3 levels (3.6 pmol/L), which was significantly lower than the level in the high T3 group (5.6 pmol/L). A negative correlation was found between the development of PAH and echo levels to fT3 levels (r_(s)_ = −0.538, r_(s)_ = −0.701, *p* ≤ 0.05; Table 2). Furthermore, the miRNA-21 levels in the PAH group were higher than the levels in the high T3 group (Figure 6). Together, these results supported our in vitro study, which suggested that there is a connection between miRNA-21 levels to fT3 levels. Finally, we examined whether there is a correlation between the anti-fibrotic fT3 level and the MRSS skin scores. We found a medium negative correlation between these two parameters; however, it did not reach statistical significance (r_(s)_ = −0.311, *p* = 0.104; Table 2). Nevertheless, among all patients, six (33%) had MRSS values >15. In support of our assumption, five of these patients belonged to the low T3 group. fT3 levels might be affected by the patients’ age and sex. Among our patients, there was only one male; therefore, the effect of sex on fT3 is not relevant to this study or its results (Table 1). We found a negative correlation between age and fT3 level; however, there was no correlation between age and miRNA-21 (Table 2). This suggests that age may be involved in mediating the connection between fT3 and miRNA-21 levels. However, an additional parameter is probably also involved. Our in vitro study suggested that D3 is the missing link between them. While fT3 and miRNA-21 were previously found in the circulation, D3 is a plasma membrane enzyme, and we could not evaluate its level in the patients’ skin to check this point.

We were impressed by the significant correlation between the development of PAH/increased echo (that characterizes PAH) to the levels of fT3 (Table 2). The blood samples used in this study were donated to the Biobank about 4 years ago, and we checked whether any patients had developed PAH since then. We found that one patient in the low T3 group developed PAH, and the echo level of another patient increased dramatically. This suggests that the development of PAH is a linking factor between high miRNA-21 levels and reduced fT3. The assumption regarding the connection between PAH, miRNA-21, D3, and T3 should be further studied in the future.

## 3. Discussion

In this study, we constructed a biological model to examine the effect of ECM with fibrotic characteristics on the phenotype of DF and their αvβ3 integrin expression. This model was created to study the function of DF in a fibrotic microenvironment, as found in SSc and other fibrosis-related diseases. Using this model, we examined the connection between the THs binding site of αvβ3 and fibrosis-related pathways. To study this relationship, an ECM was constructed by exposing DF to TGFβ (fibrotic-ECM). The effect of the fibrotic-ECM on DF was demonstrated and showed the induction of a vicious cycle: TGFβ-treated DF differentiated into MF and produced fibrotic-ECM that induced DF differentiation to MF, and so on. DF differentiation to MF was accompanied by increased expression of αvβ3 integrin, the pro-fibrotic miRNA-21, and the D3 enzyme. Lastly, we demonstrated that tetrac prevented DF differentiation into MF as well as the increased miRNA-21 and D3 levels found in the MF. Since D3 enzyme degrades T3, these results suggest a link between miRNA-21 and T3 levels. This link was supported by the negative correlation between fT3 and miRNA-21 levels found in SSc patients’ circulation.

To produce ECM, normal-ECM (−TGFβ) was secreted by DF and fibrotic-ECM was secreted by DF triggered by TGFβ in two different models. In the +TGFβ model, the ECM was secreted during constant exposure to exogenous TGFβ. The +−TGFβ model (the autologous model) demonstrated that cells that undergo primary stimulation with TGFβ could continue to secrete pro-fibrotic factors without additional external stimulation. This supports a previous observation, which demonstrated that fibrosis is a self-reinforcing process [36]. Characterization of the secreted matrices showed that both fibrotic-ECMs contained increased levels of proteins and were enriched with collagen-I, elastin, fibronectin and TGFβ compared to the normal-ECM. These proteins are similar to those found in fibrotic areas and to the composition of the fibrotic skin of SSc patients [8,9,19,37].

The fibrotic-ECMs we created induced DF differentiation to MF. This could have been triggered by different factors in the ECM. The level of proteins, especially collagen, affects matrix stiffness [37,38]. Previous publications showed a critical role for ECM stiffening in early MF activation in fibrosis [39]. Integrins can sense ECM stiffness [39] and affect the DF phenotype. The higher collagen-I levels found in our fibrotic-ECMs suggest that they were stiffer than the normal-ECM and this may have contributed to the DF differentiation to MF. Furthermore, another study showed that the presence of extracellular elastin increases the expression of MF biomarkers, such as αSMA and collagen-I [40]. Therefore, the increased elastin levels in our fibrotic-ECMs may have also contributed to the MF differentiation. The ECM is also a reservoir for growth factors, including TGFβ, which is mostly found embedded in the ECM [19]. We found a trend of increased TGFβ levels in the fibrotic-ECMs compared to the levels in the normal-ECM and increased SMAD3 phosphorylation in DF cultured on the fibrotic-ECMs. These results suggest that the TGFβ pathway was activated in these cells. We also found that DF cultured on our fibrotic-ECMs express higher αv levels and MMP-9 activity that can release TGFβ from the ECM. In agreement with these observations, integrin β3 was previously found to increase MMP-9 levels [41]. Furthermore, previous publications showed increased MMP-9 levels in DF of SSc patients, which correlated with the pathological skin involvement [42], increased αv integrin levels in MF [43] and increased αvβ3 integrin levels in SSc DF [44].

The results of this study showed increased αvβ3 integrin expression in DF cultured on fibrotic-ECMs. Previous studies have demonstrated that DF differentiation into MF involves αvβ3 integrin-mediated focal adhesion kinase signaling [39]. αvβ3 integrin is expressed in minimal amounts in normal tissues [45] and higher levels are found in tumors and fibrotic areas [46]. Together, this suggests that αvβ3 integrin may be a suitable target for treating fibrosis, as previously suggested [47]. Interestingly, since αvβ3 is also a cell surface receptor for THs [24], its high expression in MF at fibrotic areas could suggest that THs affect the fibrotic process through this integrin. To test this hypothesis, we added tetrac to DF cultured on the different ECMs and found that it prevented DF differentiation to MF. We chose to use the αvβ3 ligand tetrac and not T3 or its precursor T4, since tetrac is a powerful ligand of αvβ3, yet only has a very low grade of thyromimetic activity [48]. A previous publication showed that the addition of tetrac inhibited cancer cell growth [49]. Yet, to the best of our knowledge, the current study is the first to demonstrate that tetrac prevents DF differentiation into MF. We also found that tetrac inhibited αvβ3 expression in DF cultured on fibrotic-ECM. Previous observations showed that in several cancer cells, T3 and T4 increased the expression of αvβ3 integrin [50,51,52]. Furthermore, similar to our results, RGD, neutralizing αvβ3 antibodies, and tetrac reduced the expression of αvβ3 in the cancer cells [52]. Together, this suggests that tetrac prevented T4/T3 binding to the integrin, as well as its expression.

We also demonstrated that DF cultured on the fibrotic-ECM expressed higher levels of miRNA-21 and the T3 degrading enzyme D3. Tetrac reduced both miRNA-21 and D3 levels. These results support the ability of tetrac to increase the level of T3 in cells exposed to it and link miRNA-21 to D3. The ability of tetrac to decrease miRNA-21 levels was previously demonstrated in cancer [49,53]. miRNA-21 positively regulates the level of D3 via its suppressor, *GRHL3* [14], affording a possible explanation for the increased levels of D3 found in our system. The ability of tetrac to reduce D3 levels suggests that the THs binding site of αvβ3 integrin also affects fibrosis by modulating T3 levels. We validated the connection between miRNA-21 and fT3 by showing that SSc patients with relatively low fT3 levels have higher miRNA-21 levels than patients with higher fT3 levels. While miRNA-21 and T3 can be found in the circulation, D3 is a plasma membrane enzyme, and we could not evaluate its level in the patients’ skin to check the connection between its level to T3 and miRNA-21 levels. The SSc patients included patients with PAH, which is a leading cause of mortality in SSc [54]. The patients with PAH had low fT3 levels and high miRNA-21 levels. These results support previous studies that showed that PAH is associated with hypothyroidism [55] and that PAH patients have higher circulating miRNA-21 levels than SSc patients without PAH [56]. These publications strengthen the reliability of our results and the link between the levels of the pro-fibrotic miRNA-21, and T3, and suggest that a larger group of PAH patients should be further investigated to better understand the relationship between miRNA-21, D3, and T3.

The results of this study demonstrate that tetrac inhibits fibrosis by preventing DF differentiation to MF and hence the production of the fibrotic-ECM, by inhibiting the αvβ3-miRNA-21-D3-T3 pathway, and by reducing the levels of αvβ3.

The connection between altered THs levels and fibrosis was previously described [29,30,31]. Several studies showed a connection between the development of SSc, autoimmune thyroiditis, and hypo/hyperthyroidism [29,30]. Interestingly, Gionfra et al. suggested the possibility that inhibitors of αvβ3 integrin might represent a future therapeutic tool against liver cancer and its complications, such as liver fibrosis [51]. The current study supports the observations of a connection between the level of THs to the development of fibrosis, specifically in SSc patients. The results presented here provide a mechanism that explains these observations. It should be noted that in most of our patients, the fT3 level was in the low normal range. These results suggest that very subtle changes in fT3 levels can modulate the fibrosis process.

Several mechanisms can explain our observations of the anti-fibrotic effects of tetrac. Tetrac binding to the αvβ3 integrin might have inhibited the release of TGFβ from the ECM by this integrin. Yet, there is controversy over whether this integrin can release TGFβ. While some articles suggest that the integrin releases TGFβ, others claim that it cannot. [57,58]. Furthermore, the THs binding site of αvβ3 is located close to the RGD recognition site of this integrin. The RGD sequence is found in many ECM proteins, among which are fibronectin and the LAP of TGFβ precursor. It might be that the co-binding of tetrac and RGD-containing proteins (like fibronectin) to αvβ3, activated downstream signaling that inhibited the TGFβ pathway. It might also be that T4 or another ligand binds to the integrin to facilitate activation of the TGFβ pathway and that tetrac prevented this effect. Indeed, one study showed that integrin β3 could activate the TGFβ pathway [41]. Another study showed that tetrac affected the TGFβ pathway in oral cancer cells [59], suggesting that it might indeed have modulated integrin αvβ3 downstream signaling. However, in our model, DF cultured on normal-ECM also expressed αvβ3. Although those cells expressed lower levels of the integrin than cells cultured on fibrotic-ECM, we would have expected tetrac to affect the TGFβ pathway in those cells as well, which did not happen. This reinforces the assumption that binding of the RGD ligand found in the fibrotic-ECM to the integrin affected the tetrac downstream signaling.

The limitations of this study include the small group of patients evaluated, the absence of data regarding D3 and T3 levels in the patients’ skin, and the fact that we did not compare the SSc data to data obtained from healthy people. Furthermore, while D3 inactivates T3, D1 and D2 activate T4 to T3 [60]. In future studies, it will be interesting to evaluate the levels of these enzymes and thyroid hormone derivatives such as tetrac in SSc patients to understand the connection between them and the development of SSc and complications like PAH.

## 4. Materials and Methods

### 4.1. Isolation of Dermal Fibroblasts (DF)

Cells were isolated from the skin tissue of healthy patients who underwent abdominoplasty in the Plastic Surgery Department of Meir Medical Center. Skin biopsies were placed on a sterile plate with 1% PBS antibiotics (Pen-Strep-Nystatin (PSN, 03-032-1B), Biological Industries, Kibbutz Beit-Haemek, Israel). The fat was removed with scissors, and the biopsies were cut into small pieces using a 4 mm seamless, disposable biopsy punch. The biopsies were then transferred into a tube with 7 mL collagenase mix (0.02 g collagenase type 2 (17101-015, GIBCO, Waltham, MA, USA), 0.001 g DNAse (11284932001, Sigma Aldrich, Rehovot, Israel), 2 mL TrypsinX10 (03-046-5B, Biological Industries, Kibbutz Beit-Haemek, Israel), and 0.1 g BSA (160069 MP Biomedical, Santa Ana, CA, USA), in 20 mL Dulbecco’s Modified Eagle Medium (DMEM, 01-055-1A, Biological Industries, Kibbutz Beit-Haemek, Israel)), vortexed for 30 s and placed in a rotating incubator at 37 °C for 1.5 h. The enzymatic reaction was stopped by adding 1:1 serum-containing medium to the tubes. The tubes were centrifuged at 1700 rpm for 8 min and incubated in DMEM (01-055-1A, Biological Industries), containing 20% fetal bovine serum (FBS, 04-007-1A, Biological Industries, Kibbutz Beit-Haemek, Israel), 2 mM glutamine (03-020-1B, Biological Industries), and PSN (03-032-1B Biological Industries, Kibbutz Beit-Haemek, Israel) in 100 mm × 20 mm Corning culture plates. Over 2 to 3 weeks, the tissue residues were disposed of, and the DF remained on the plates with a similar medium containing 10% FBS (04-007-1A, Biological Industries, Kibbutz Beit-Haemek, Israel).

### 4.2. Immunocytochemistry (ICC)

DF, mesenchymal stem cells (MSC), and lung carcinoma epithelial cells were tested for expression of epithelial (keratin) and mesenchymal (vimentin) markers. Furthermore, DF cultured on ECMs were tested for the expression of αvβ3 integrin. Cells were fixed in 24-well plates with formaldehyde (0006450323F1, Bio-Lab, Ltd., Jerusalem, Israel). Endogenous peroxidase activity was quenched in PBS-H_2_O_2_ 1% (3 mL H_2_O_2_ 3% + PBSx1 FLORIS). Cells were blocked using goat serum (04-009-1A, Biological Industries, Kibbutz Beit-Haemek, Israel) and incubated with a primary antibody (rabbit anti-Vimentin, rabbit anti-keratin, or rabbit anti-αvβ3 integrin; details in Table 3), at 4 °C overnight. Cells were washed and incubated with horseradish peroxidase-labeled polymer conjugated to a secondary antibody (ZUC053006 Zytomed, Berlin, Germany), washed, and developed with the 3-amino-9-ethylcarbazole (AEC) substrate buffer (ACG500, ScyTek Laboratories, Logan, UT, USA). Cell nuclei were stained with hematoxylin (MFCD00147088, Merck, Darmstadt, Germany).

### 4.3. Adipogenesis/Osteogenesis Differentiation

The multi-potency of the cells was evaluated by assessing their capacity to differentiate into adipocytes or osteoblasts using StemPro adipogenesis and osteogenesis differentiation kits (A1007001 and A1007201, respectively, GIBCO, Waltham, MA, USA), according to the manufacturer’s instructions. Cells were cultured in 24-well plates for 14–21 days with adipogenesis/osteogenesis differentiation media. Adipogenic and osteogenic differentiations were demonstrated using Sudan IV (85-83-6, Sigma Aldrich, Rehovot, Israel) and Alizarin Red (A5533, Sigma Aldrich, Rehovot, Israel) staining, respectively.

### 4.4. Extracellular Matrix Preparation

DF were cultured in 24-well plates under the following conditions: (1) without TGFβ ((100-21C-21, PeproTech, Rehovot, Israel), to create “normal-ECM”); (2) with TGFβ (to create “fibrotic-ECM” during constitutive activation of TGFβ); and (3) one day with TGFβ and another 3 days without TGFβ (to create “fibrotic-ECM” in a model that imitates autologous activation). Ninety-six hours later, cells were removed by washing the wells with: (a) double distilled water (DDW) for 10 min in an incubator; (b) NH_4_OH (1336-21-6, Merck), 0.3%, diluted in DDW) for 5 min at room temperature; and (c) DDW briefly. The ECM was left in the wells. Next, one of the following was undertaken: (1) the ECM was collected from the wells using DTT buffer (0.1M DTT, SDS 10% (01-890-1B, Biological Industries, Kibbutz Beit-Haemek, Israel), tris (0020092309100, Bio-Lab, Jerusalem, Israel), pH 8 1M, DDW). The samples were precipitated with acetone, and the ECM proteins were assessed for collagen-I, elastin, fibronectin, and TGFβ levels using Western blot. (2) The ECM was stained as described in Section 2.5.

### 4.5. ECM Histochemical Staining

The ECM proteins left in the wells were stained using 3 different procedures, as follows. (1) General protein staining: Coomassie blue was added to the wells for 2 min and then washed with DDW. (2) Collagen staining: Proteins were fixed with 10% formaldehyde (0006450323F1, Bio-Lab, Ltd., Jerusalem, Israel) for 20 min, washed with 70% ethanol (1009832500, Mercury, Rosh-Ha’ayin, Israel) and DDW, incubated in aniline acid (2.5 g aniline blue, 2 mL glacial acetic acid (2619938, Merck, 100 mL DDW)) for 10 min, washed with DDW, differentiated in 1% acetic acid for 2 min, and quickly washed with ethanol absolute (1009832500, Mercury, Rosh-Ha’ayin, Israel). (3) Elastin staining: Proteins were fixed with 10% formaldehyde (0006450323F1, Bio-Lab, Ltd., Jerusalem, Israel), washed gently with DDW, and incubated in fresh acid orcein (1 g orcein, 1400-62-0, Merck; 100 mL 70% ethanol (1009832500, Mercury, Rosh-Ha’ayin, Israel) and 1 mL 25% HCl) for 48 h. Wells were dehydrated with ethanol absolute (1009832500, Mercury, Rosh-Ha’ayin, Israel).

### 4.6. Cell Culture

DF were cultured in DMEM (01-055-1A, Biological Industries, Kibbutz Beit-Haemek, Israel) supplemented with 10% FBS (04-007-1A, Biological Industries), 2 mM glutamine (03-020-1B, Biological Industries, Kibbutz Beit-Haemek, Israel), and antibiotics (100 U/mL penicillin, 100µg/mL streptomycin, nystatin, 03-032-1B, Biological Industries, Kibbutz Beit-Haemek, Israel). Cells were incubated at 37 °C in 5% CO_2_ and split twice per week using trypsin (03-052-1A, Biological Industries, Kibbutz Beit-Haemek, Israel), upon reaching 80% confluence. We used DF in the experiments until passage 8.

### 4.7. Cell Count

Trypan-blue (EBT-001 EnTek, Lebanon, OR, USA) was mixed in a 1:1 ratio with the cells. Then, the cells were counted using a countess automatic cell counter (Invitrogen, Waltham, MA, USA). Live cells were unstained, and dead cells assimilated the dye.

### 4.8. Protein Extraction

DF were lysed in a RIPA buffer (ab156034, Abcam, Cambridge, UK) for 10 min on ice and centrifuged (15 min, 15,000 rpm, 4 °C). Protein levels were determined with a BCA protein assay kit (23225 Pierce, Rockford, IL, USA) according to the manufacturer’s instructions.

### 4.9. Western Blotting

To assess the levels of collagen-I, elastin, αSMA, cyclin-D1, αv, β3, phospo-SMAD3, D3, TGFβ, fibronectin, and tubulin in DF or in the ECM, protein lysates were mixed (1:5) with sample buffer (250 mM Tris–HCl (0020092309100, Bio Lab, Ltd., Jerusalem, Israel), pH 6.8, 400 mM DTT (A39255, Thermo Scientific, Waltham, MA, USA), 140 mM SDS (01-890-1B, Biological Industries), 60% glycerin, 0.02% bromophenol blue (115-39-9, Merck), with/without beta-mercaptoethanol (M6250, Sigma Aldrich, Rehovot, Israel) and denatured (or not, depending on the antibody) for 10 min at 65 °C. Proteins (10–25 μg) from each sample were separated using electrophoresis on SDS-PAGE and transferred to a PVDF membrane (IPVH00010, Millipore, Burlington, MA, USA).

Transfer efficiency was validated using Ponceau staining (Sigma Aldrich, Rehovot, Israel). After blocking the non-specific binding sites with 5% milk powder (232100, BD, San Jose, CA, USA) in Tris-buffered saline (TBS) (0020092309100, Bio-Lab, Ltd., Jerusalem, Israel) containing 0.1% Tween (8.22184.0500, Merck Germany, TBS-T), the membrane was incubated with the primary antibodies at 4 °C, overnight (see Table 3 for antibody details). Primary antibodies were rinsed with TBS-Tween and TBS. Bound antibodies were visualized using a peroxidase-conjugated secondary antibody (Table 3), followed by enhanced chemiluminescence detection (WBKLS0500, Mercury, Rosh-Ha’ayin, Israel). Optical densities were visualized and measured in arbitrary units using an LAS3000 Image reader. Proteins isolated from cells were normalized to tubulin. As the ECM does not contain tubulin, equal amounts of buffer were added to the extracted proteins and loaded into the gels, and the protein levels were normalized relative to the loaded volume. Protein levels were assessed using the Multi-gauge V3.0 program (Fujifilm, Tokyo, Japan).

### 4.10. Gelatin Zymography

Cell secretomes (20–40 µL) were electrophoresed at non-reducing conditions in 10% polyacrylamide gel, containing 1 mg/mL gelatin type 1 (G2500, Sigma Aldrich, Rehovot, Israel). Gels were washed in 2.5% Triton X-100 (Sigma Aldrich, Rehovot, Israel) and incubated overnight in 50 mM Tris-HCl (pH 7.5) and 5 mM CaCl_2_. Coomassie blue (1610786, Bio-Rad) staining followed by destaining (20% methanol (1060092500, Mercury, Rosh-Ha’ayin, Israel), 7% acetic acid (2619938, Merck in DDW) enabled the visualization of clear lysis zones contrasted on the blue background. A molecular weight standard was used to validate the size of the MMP. The optical densities of the clear zones were measured in arbitrary units using a LAS3000 Image reader. Results were normalized to background values using the Multi-gauge V3.0 program (Fujifilm).

### 4.11. RNA Extraction

RNA was extracted from DF, using miRNeasy Tissue/Cells Advanced Mini Kit (217684, QIAGEN, Hilden, Germany) according to the manufacturer’s instructions, and frozen at −80 °C.

### 4.12. RT cDNA Synthesis

Extracted RNA was converted to cDNA using TaqMan Advanced miRNA cDNA Synthesis Kit (Applied Biosystems, Waltham, MA, USA), according to the manufacturer’s instructions.

### 4.13. Real-Time Quantitative PCR

PCR reaction was conducted using TaqMan Fast Advanced Master Mix (4444556, Applied Biosystems) and TaqMan Advanced miRNA Assay (A25576, Applied Biosystems) for miRNA-21 (hsa-21-5p), according to the manufacturer’s instructions (A28007, Applied Biosystems). miRNA-16 (hsa-16-5p) served as a normalizing miRNA.

### 4.14. Analyzing the Effect of TGFβ on DF Differentiation into MF, and the Cells’ Ability to Create Fibrotic ECM

DF were cultured in 24-well plates with TGFβ (10 ng/mL) for 72 h. The control cells were cultured using a medium without TGFβ (100-21C-21, PeproTech, Rocky Hill, NJ, USA). Cells were then harvested, and one of the following was performed: (1) Proteins were extracted and analyzed for collagen-I, elastin, αSMA, cyclin-D1, and tubulin levels (Western blot), (2) the number of live and dead cells was counted (using an automatic cell counter and trypan-blue). The ability of the cells to create a fibrotic matrix was evaluated, as described in Section 4.2 and Section 4.9.

### 4.15. Analyzing the Effect of ECM on DF

Normal or fibrotic ECMs were prepared as described in Section 2.4. DF were cultured on the ECMs for 48–72 h with or without 0.5 μM tetrac (T3787, Sigma Aldrich, Rehovot, Israel) dissolved in dimethyl sulfoxide (DMSO)-KOH propylene glycol 1 mM (1% DMSO). As a control, the cells were cultured for the same period in medium with or without DMSO-KOH in the same concentration as the tetrac (1:200,000). Cells were later harvested and analyzed for the following: (1) cell number and death (using an automatic cell counter and trypan-blue); (2) levels of collagen-I, elastin, αSMA, cyclin-D1, αv, β3, phospo-SMAD3, and D3 (using protein extraction and Western blot); (3) the activity of secreted MMPs (using gelatin zymography); and (4) the level of hsa-21-5p (using RNA extraction and qPCR; hsa-16-5p served to normalize results).

### 4.16. Ethical Approval

The study was conducted according to the guidelines of the Declaration of Helsinki, and approved by the Institutional Ethics Committee of Meir Medical Center (Helsinki, protocol codes: for isolation of fibroblasts: 222-20-MMC, date of approval 7 March 2021 and for blood samples 116-17 MMC 5 June 2017). Informed consent was obtained from all participants.

### 4.17. Patient Data

Blood samples of 18 SSc patients (2013 EULAR/ACR classification) were collected from the Biobank of Meir Medical Center. Subgroup classification included 11 SSc patients with and 7 without pulmonary arterial hypertension (PAH) (diagnosed by echocardiogram and right heart catheterization). All blood samples collected were from patients at least two years after disease onset. The clinical features of the patients are described in Table 1.

### 4.18. Biobank

The autoimmune laboratory at Meir Medical Center in Israel manages a rheumatology biobank that contains plasma, serum, and blood cells collected from rheumatologic patients since April 2018. Blood samples are collected from participants who have previously consented and authorized the use of their samples for research purposes. Blood samples are separated into serum, plasma, and cells, and kept at −80 °C.

### 4.19. Statistical Analysis

Cell culture experiments were repeated separately 4-8 times. A paired student t-test was used to analyze the differences between the cohorts. Spearman’s Rho correlation was used to evaluate the association between fT3 to the research variables MRSS, PAH and miRNA-21. The non-parametric Mann–Whitney *U* test was used to compare miRNA-21 levels in the different SSc patient groups. The statistical analyses were performed using SPSS/PC statistical software, version 27.0 (IBM Corp., Armonk, NY, USA). An effect was considered significant when the *p*-value was ≤0.05.

## 5. Conclusions

This study shows that manipulating the THs binding site of αvβ3 integrin affects the development of fibrosis and suggests that blocking this site by tetrac may delay the development of fibrosis.

## Figures and Tables

**Table 1:** Clinical characteristics of the SSc patients.

Characteristics	Low T3 PAH	All Low T3 SSc + PAH	High T3 SSc
	*n* = 7	*n* = 12	*n* = 6
Demographics			
Age (years)	67 ± 9 *	60 *	40 ± 16
Female (% (number of males))	86 (1)	91.7 (1)	100
Clinical			
Diffuse vs. local SSc (%)	57	66	83
Echo-pulmonary arterial pressure (mPAP, mmHg)	68 ± 19 *	54.6 ± 22 *	27.3 ± 3.6
Average FT3 level (pmol/L) (normal range 3.1–6.8 pmol/L)	3.6 ± 0.6 *	3.7 ± 0.5 *	5.6 ± 1.4
miRNA-21	0.56 ± 0.46 *	0.47 + 0.46 *	0.12 + 0.15

* Significantly different than the high T3 group.

**Table 2:** Correlations between fT3 and miRNA-21, PAH, and MRSS.

Variables		r_(s)XY_	*p*-Value	*n*
X	Y			
fT3	miRNA-21	−0.391	0.0505	18
fT3	MRSS	−0.311	0.104	18
fT3	PAH	−0.538	0.010	18
fT3	Echo	−0.701	0.001	18
fT3	Age	−0.424	0.036	18
miRNA-21	Age	0.243	0.329	18

**Table 3:** List of antibodies.

Target	Source	Isotype	Company	Catalog No.	Dilution	Hybridization Conditions
Vimentin	Rabbit	mAb	Cell Signaling Technology (Danvers, MA, USA)	M0725	1:100	BSA 3%
Pan Cytokeratin	Mouse	mAb	BIOCARE (Pacheco, CA, USA)	CM 011 A, B, C	1:1	As is
Collagen-I	Rabbit	Polyclonal Ab	Abcam (Cambridge, UK)	ab34710	1:1000	BSA 5%
Elastin	Rabbit	Polyclonal Ab	Proteintech	15257-1	1:2500	BSA 5%
Fibronectin	Rabbit	mAb	Abcam (Cambridge, UK)	AB32419	1:1000	Milk 5%
αSMA	Mouse	mAb IgG2a	Abcam (Cambridge, UK)	ab7817	1:2000	BSA 5%
Cyclin-D1	Rabbit	Polyclonal Ab	Cell Signaling Technology (Danvers, MA, USA)	#2922	1:1000	BSA 5%
Integrin αv	Rabbit	Polyclonal Ab	Cell Signaling Technology (Danvers, MA, USA)	#4711	1:1000	BSA 5%
Integrin β3	Rabbit	Polyclonal Ab	Santa Cruz Biotechnology	SC14009	1:200	BSA 5%
Integrin αvβ3	Rabbit	Polyclonal Ab	ABBIOTEC (Rosemont, IL, USA)	251672	1:200	BSA 3%
Phospho-SMAD3	Rabbit	mAb IgG	Cell Signaling Technology (Danvers, MA, USA)	#9520	1:1000	BSA 5%
D3	Rabbit	Polyclonal Ab	Novus (Centennial, CO, USA)	NBP1-05767	1:2000	BSA 5%
TGFβ-I	Rabbit	Polyclonal Ab	Sigma Aldrich (Rehovot, Israel)	HPA008612	1:1000	BSA 5%
α-Tubulin	Mouse	mAb IgG1	Sigma Aldrich Rehovot, Israel)	T5168	1:4000	Milk 5%
Peroxidase conjugated anti-mouse	Goat	Polyclonal Ab	Jackson Immuno-Research (West Grove, PA, USA)	115035003	1:20,000	Milk 1%
Peroxidase conjugated anti-Rabbit	Goat	Polyclonal Ab	Jackson Immuno-Research (West Grove, PA, USA)	111035003	1:10,000	Milk 1%

## Data Availability

No additional data were created.

## Abbreviations

αSMA	alpha-smooth muscle actin
D3 (protein)	*DIO3* (gene) deiodinase-type-3
DDW	double distilled water
DF	dermal fibroblasts
DMEM	Dulbecco’s Modified Eagle Medium
ECM	extracellular matrix
fT3	free T3
ICC	immunocytochemistry
LAP	latency-associated peptide
MF	myofibroblasts
MMP	matrix metalloproteinase
MRSS	modified Rodnan skin score
MSC	mesenchymal stem cells
PAH	pulmonary arterial hypertension
pSMAD3	phosphorylated-Smad3
RGD	arginine-glycine-aspartic
SSC	systemic sclerosis
T3	triodothyronine
T4	L-thyroxine
TBS	tris-buffered saline
Tetrac	tetraiodothyroacetic acid
TGFβ	transforming growth factor β
THs	thyroid hormones

## References

[1] Asano Y. (2018). Systemic sclerosis. J. Dermatol..

[2] Knobler R., Moinzadeh P., Hunzelmann N., Kreuter A., Cozzio A., Mouthon L., Cutolo M., Rongioletti F., Denton C., Rudnicka L. (2017). European Dermatology Forum S1-guideline on the diagnosis and treatment of sclerosing diseases of the skin, Part 1: Localized scleroderma, systemic sclerosis and overlap syndromes. J. Eur. Acad. Dermatol. Venereol..

[3] Strong A.L., Rubin J.P., Kozlow J.H., Cederna P.S. (2019). Fat Grafting for the Treatment of Scleroderma. Plast. Reconstr. Surg..

[4] Khanna D., Lin C.J.F., Furst D.E., Goldin J., Kim G., Kuwana M., Allanore Y., Matucci-Cerinic M., Distler O., Shima Y. (2020). Tocilizumab in systemic sclerosis: A randomised, double-blind, placebo-controlled, phase 3 trial. Lancet Respir. Med..

[5] Distler O., Cozzio A. (2016). Systemic sclerosis and localized scleroderma—Current concepts and novel targets for therapy. Semin. Immunopathol..

[6] Dees C., Chakraborty D., Distler J.H.W. (2021). Cellular and molecular mechanisms in fibrosis. Exp. Dermatol..

[7] Maldonado H., Hagood J.S. (2021). Cooperative signaling between integrins and growth factor receptors in fibrosis. J. Mol. Med..

[8] Liakouli V., Cipriani P., Di Benedetto P., Ruscitti P., Carubbi F., Berardicurti O., Panzera N., Giacomelli R. (2018). The role of extracellular matrix components in angiogenesis and fibrosis: Possible implication for Systemic Sclerosis. Mod. Rheumatol..

[9] Birembaut P., Cahuzac P., Delhomme H., Caron Y., Labat-Robert J., Robert L., Kalis B. (1982). Distribution of fibronectin in the skin of patients with scleroderma. Ann. Dermatol. Venereol..

[10] van Caam A., Vonk M., van den Hoogen F., van Lent P., van der Kraan P. (2018). Unraveling SSc Pathophysiology; The Myofibroblast. Front. Immunol..

[11] Sivakumar P., Kitson C., Jarai G. (2019). Modeling and measuring extracellular matrix alterations in fibrosis: Challenges and perspectives for antifibrotic drug discovery. Connect. Tissue Res..

[12] Darby I.A., Laverdet B., Bonté F., Desmouliere A. (2014). Fibroblasts and myofibroblasts in wound healing. Clin. Cosmet. Investig. Dermatol..

[13] Zhu H., Luo H., Li Y., Zhou Y., Jiang Y., Chai J., Xiao X., You Y., Zuo X. (2013). MicroRNA-21 in scleroderma fibrosis and its function in TGF-β-regulated fibrosis-related genes expression. J. Clin. Immunol..

[14] Di Girolamo D., Ambrosio R., De Stefano M.A., Mancino G., Porcelli T., Luongo C., Di Cicco E., Scalia G., Del Vecchio L., Colao A. (2016). Reciprocal interplay between thyroid hormone and microRNA-21 regulates hedgehog pathway-driven skin tumorigenesis. J. Clin. Investig..

[15] Baqui M., Botero D., Gereben B., Curcio C., Harney J.W., Salvatore D., Sorimachi K., Larsen P.R., Bianco A.C. (2003). Human type 3 iodothyronine selenodeiodinase is located in the plasma membrane and undergoes rapid internalization to endosomes. J. Biol. Chem..

[16] Alonso-Merino E., Orozco R.M., Ruíz-Llorente L., Martínez-Iglesias O.A., Velasco-Martín J.P., Montero-Pedrazuela A., Fanjul-Rodríguez L., Contreras-Jurado C., Regadera J., Aranda A. (2016). Thyroid hormones inhibit TGF-β signaling and attenuate fibrotic responses. Proc. Natl. Acad. Sci. USA.

[17] Shumnalieva R., Kachakova D., Shoumnalieva-Ivanova V., Miteva P., Kaneva R., Kolarov Z., Monov S. (2019). P109 Expression levels of miR-21 and miR-29 in the serum of systemic sclerosis patients. Ann. Rheum. Dis..

[18] Wolska-Gawron K., Bartosińska J., Rusek M., Kowal M., Raczkiewicz D., Krasowska D. (2020). Circulating miRNA-181b-5p, miRNA-223-3p, miRNA-210-3p, let 7i-5p, miRNA-21-5p and miRNA-29a-3p in patients with localized scleroderma as potential biomarkers. Sci. Rep..

[19] Frangogiannis N. (2020). Transforming growth factor-β in tissue fibrosis. J. Exp. Med..

[20] Song K.H., Cho S.J., Song J.Y. (2016). αvβ1 integrin as a novel therapeutic target for tissue fibrosis. Ann. Transl. Med..

[21] Brown N.F., Marshall J.F. (2019). Integrin-Mediated TGFβ Activation Modulates the Tumour Microenvironment. Cancers.

[22] Liu Z., Wang F., Chen X. (2008). Integrin α_v_β_3_-Targeted Cancer Therapy. Drug Dev. Res..

[23] Nishimura S.L. (2009). Integrin-mediated transforming growth factor-beta activation, a potential therapeutic target in fibrogenic disorders. Am. J. Pathol..

[24] Bergh J.J., Lin H.Y., Lansing L., Mohamed S.N., Davis F.B., Mousa S., Davis P.J. (2005). Integrin αVβ3 contains a cell surface receptor site for thyroid hormone that is linked to activation of mitogen-activated protein kinase and induction of angiogenesis. Endocrinology.

[25] Nappi A., Murolo M., Sagliocchi S., Miro C., Cicatiello A.G., Di Cicco E., Di Paola R., Raia M., D’Esposito L., Stornaiuolo M. (2021). Selective Inhibition of Genomic and Non-Genomic Effects of Thyroid Hormone Regulates Muscle Cell Differentiation and Metabolic Behavior. Int. J. Mol. Sci..

[26] Liu Y.C., Yeh C.T., Lin K.H. (2019). Molecular Functions of Thyroid Hormone Signaling in Regulation of Cancer Progression and Anti-Apoptosis. Int. J. Mol. Sci..

[27] Cohen K., Abadi U., Hercbergs A., Davis P.J., Ellis M., Ashur-Fabian O. (2018). The induction of myeloma cell death and DNA damage by tetrac, a thyroid hormone derivative. Endocr. Relat. Cancer.

[28] Cody V., Davis P.J., Davis F.B. (2007). Molecular modeling of the thyroid hormone interactions with αvβ3 integrin. Steroids.

[29] Jabbar A., Pingitore A., Pearce S.H.S., Zaman A., Iervasi G., Razvi S. (2017). Thyroid hormones and cardiovascular disease. Nat. Rev. Cardiol..

[30] Fallahi P., Ruffilli I., Giuggioli D., Colaci M., Ferrari S.M., Antonelli A., Ferri C. (2017). Associations between Systemic Sclerosis and Thyroid Diseases. Front. Endocrinol..

[31] Zhou J., Tripathi M., Ho J.P., Widjaja A.A., Shekeran S.G., Camat M.D., James A., Wu Y., Ching J., Kovalik J.-P. (2022). Thyroid Hormone Decreases Hepatic Steatosis, Inflammation, and Fibrosis in a Dietary Mouse Model of Nonalcoholic Steatohepatitis. Thyroid.

[32] Yu G., Tzouvelekis A., Wang R., Herazo-Maya J.D., Ibarra G.H., Srivastava A., Werneck-De-Castro J.P., DeIuliis G., Ahangari F., Woolard T. (2018). Thyroid hormone inhibits lung fibrosis in mice by improving epithelial mitochondrial function. Nat. Med..

[33] Ding D.C., Shyu W.C., Lin S.Z. (2011). Mesenchymal stem cells. Cell Transpl..

[34] Tobi D., Krashin E., Davis P.J., Cody V., Ellis M., Ashur-Fabian O. (2022). Three-Dimensional Modeling of Thyroid Hormone Metabolites Binding to the Cancer-Relevant αvβ3 Integrin: In-Silico Based Study. Front. Endocrinol..

[35] Khanna D., Furst D.E., Clements P.J., Allanore Y., Baron M., Czirjak L., Distler O., Foeldvari I., Kuwana M., Matucci-Cerinic M. (2017). Standardization of the modified Rodnan skin score for use in clinical trials of systemic sclerosis. J. Scleroderma Relat. Disord..

[36] Wynn T.A. (2008). Cellular and molecular mechanisms of fibrosis. J. Pathol..

[37] Duca L., Blaise S., Romier B., Laffargue M., Gayral S., El Btaouri H., Kawecki C., Guillot A., Martiny L., Debelle L. (2016). Matrix ageing and vascular impacts: Focus on elastin fragmentation. Cardiovasc. Res..

[38] Deng B., Zhao Z., Kong W., Han C., Shen X., Zhou C. (2022). Biological role of matrix stiffness in tumor growth and treatment. J. Transl. Med..

[39] Santos A., Lagares D. (2018). Matrix Stiffness: The Conductor of Organ Fibrosis. Curr. Rheumatol. Rep..

[40] Blaauboer M.E., Boeijen F.R., Emson C.L., Turner S.M., Zandieh-Doulabi B., Hanemaaijer R., Smit T.H., Stoop R., Everts V. (2014). Extracellular matrix proteins: A positive feedback loop in lung fibrosis?. Matrix Biol..

[41] Rapisarda V., Borghesan M., Miguela V., Encheva V., Snijders A.P., Lujambio A., O’loghlen A. (2017). Integrin Beta 3 Regulates Cellular Senescence by Activating the TGF-β Pathway. Cell Rep..

[42] Leong E., Bezuhly M., Marshall J.S. (2021). Distinct Metalloproteinase Expression and Functions in Systemic Sclerosis and Fibrosis: What We Know and the Potential for Intervention. Front. Physiol..

[43] Conroy K.P., Kitto L.J., Henderson N.C. (2016). αv integrins: Key regulators of tissue fibrosis. Cell Tissue Res..

[44] Asano Y., Ihn H., Yamane K., Jinnin M., Mimura Y., Tamaki K. (2005). Increased expression of integrin α_v_β_3_ contributes to the establishment of autocrine TGF-β signaling in scleroderma fibroblasts. J. Immunol..

[45] Cobb D.A., de Rossi J., Liu L., An E., Lee D.W. (2022). Targeting of the α_v_β_3_ integrin complex by CAR-T cells leads to rapid regression of diffuse intrinsic pontine glioma and glioblastoma. J. Immunother. Cancer.

[46] Schnittert J., Bansal R., Storm G., Prakash J. (2018). Integrins in wound healing, fibrosis and tumor stroma: High potential targets for therapeutics and drug delivery. Adv. Drug Deliv. Rev..

[47] Yokosaki Y., Nishimichi N. (2021). New Therapeutic Targets for Hepatic Fibrosis in the Integrin Family, α8β1 and α11β1, Induced Specifically on Activated Stellate Cells. Int. J. Mol. Sci..

[48] Schmohl K.A., Nelson P.J., Spitzweg C. (2019). Tetrac as an anti-angiogenic agent in cancer. Endocr. Relat. Cancer.

[49] Gionfra F., De Vito P., Pallottini V., Lin H.-Y., Davis P.J., Pedersen J.Z., Incerpi S. (2019). The Role of Thyroid Hormones in Hepatocyte Proliferation and Liver Cancer. Front. Endocrinol..

[50] Abadi U., Weisz A., Kidron D., Katzav A., Hercbergs A., Davis P.J., Ellis M.H., Ashur-Fabian O. (2021). αvβ3 Integrin Expression and Mitogenic Effects by Thyroid Hormones in Chronic Lymphocytic Leukemia. J. Clin. Med..

[51] Godugu K., Sudha T., Davis P.J., Mousa S.A. (2021). Nano Diaminopropane tetrac and integrin αvβ3 expression in different cancer types: Anti-cancer efficacy and Safety. Cancer Treat. Res. Commun..

[52] Shinderman-Maman E., Cohen K., Weingarten C., Nabriski D., Twito O., Baraf L., Hercbergs A., Davis P.J., Werner H., Ellis M. (2016). The thyroid hormone-αvβ3 integrin axis in ovarian cancer: Regulation of gene transcription and MAPK-dependent proliferation. Oncogene.

[53] Davis P.J., Glinsky G.V., Lin H.Y., Leith J.T., Hercbergs A., Tang H.Y., Ashur-Fabian O., Incerpi S., Mousa S.A. (2014). Cancer Cell Gene Expression Modulated from Plasma Membrane Integrin αvβ3 by Thyroid Hormone and Nanoparticulate Tetrac. Front. Endocrinol..

[54] Asano Y. (2020). The Pathogenesis of Systemic Sclerosis: An Understanding Based on a Common Pathologic Cascade across Multiple Organs and Additional Organ-Specific Pathologies. J. Clin. Med..

[55] Ghamra Z.W., Dweik R.A., Arroliga A.C. (2004). Hypothyroidism and pulmonary arterial hypertension. Am. J. Med..

[56] Wuttge D.M., Carlsen A.L., Teku G., Wildt M., Rådegran G., Vihinen M., Heegaard N.H.H., Hesselstrand R. (2021). Circulating plasma microRNAs in systemic sclerosis-associated pulmonary arterial hypertension. Rheumatology.

[57] Bachmann M., Kukkurainen S. (2019). Cell Adhesion by Integrins. Physiol. Rev..

[58] Sarrazy V., Koehler A., Chow M.L., Zimina E., Li C.X., Kato H., Caldarone C.A., Hinz B. (2014). Integrins αvβ5 and αvβ3 promote latent TGF-β1 activation by human cardiac fibroblast contraction. Cardiovasc. Res..

[59] Huang C.-H., Huang T.-Y., Chang W.-J., Pan Y.-S., Chu H.-R., Li Z.-L., Unson S., Chin Y.-T., Lin C.-Y., Huang H.-M. (2020). Combined Treatment of Heteronemin and Tetrac Induces Antiproliferation in Oral Cancer Cells. Mar. Drugs.

[60] Köhrle J., Frädrich C. (2022). Deiodinases control local cellular and systemic thyroid hormone availability. Free Radic. Biol. Med..

